# High insecticide resistance mediated by different mechanisms in *Culex quinquefasciatus* populations from the city of Yaoundé, Cameroon

**DOI:** 10.1038/s41598-021-86850-7

**Published:** 2021-04-01

**Authors:** Abdou Talipouo, Konstantinos Mavridis, Elysée Nchoutpouen, Borel Djiappi-Tchamen, Emmanouil Alexandros Fotakis, Edmond Kopya, Roland Bamou, Sévilor Kekeunou, Parfait Awono-Ambene, Vasileia Balabanidou, Sofia Balaska, Charles Sinclair Wondji, John Vontas, Christophe Antonio-Nkondjio

**Affiliations:** 1 Laboratoire de Recherche Sur Le PaludismeLaboratoire de Recherche Sur Le Paludisme, Organisation de Coordination Pour la Lutte Contre les Endémies en Afrique Centrale (OCEAC), B. P. 288, Yaoundé, Cameroun; 2 Department of Animal Biology and Physiology, Faculty of Sciences, University of Yaoundé 1, P.O. Box 337, Yaoundé, Cameroon; 3 Institute of Molecular Biology and Biotechnology, Foundation for Research and Technology-Hellas, 70013 Heraklion, Greece; 4 Vector Borne Diseases Laboratory of the Research Unit Biology and Applied Ecology (VBID-RUBAE), Department of Animal Biology, Faculty of Science of the University of Dschang, Dschang, Cameroon; 5 Department of Vector Biology Liverpool School of Tropical Medicine Pembroke Place, Liverpool, L3 5QA UK; 6 Centre for Research in Infectious Disease (CRID), P.O. Box 13591, Yaoundé, Cameroun; 7 Pesticide Science Laboratory, Department of Crop Science, Agricultural University of Athens, 11855 Athens, Greece

**Keywords:** Entomology, Population dynamics

## Abstract

*Culex* mosquitoes particularly *Culex quinquefasciatus* are important arboviral and filariasis vectors, however despite this important epidemiological role, there is still a paucity of data on their bionomics. The present study was undertaken to assess the insecticide resistance status of *Cx. quinquefasciatus* populations from four districts of Yaoundé (Cameroon). All *Culex quinquefasciatus* populations except one displayed high resistance to bendiocarb and malathion with mortalities ranging from 0 to 89% while high resistance intensity against both permethrin and deltamethrin was recorded. Molecular analyses revealed high frequencies of the *ACE*-1 G119S mutation (ranging from 0 to 33%) and *kdr* L1014F allele (ranging from 55 to 74%) in all *Cx. quinquefasciatus* populations. Significant overexpression was detected for cytochrome P450s genes *CYP6AA7* and *CYP6Z10*, as well as for *Esterase A* and *Esterase B* genes*.* The total cuticular hydrocarbon content, a proxy of cuticular resistance, was significantly increased (compared to the S-lab strain) in one population. The study confirms strong insecticide resistance mediated by different mechanisms in *Cx. quinquefasciatus* populations from the city of Yaoundé. The expansion of insecticide resistance in *Culex* populations could affect the effectiveness of current vector control measures and stress the need for the implementation of integrated vector control strategies in urban settings.

## Introduction

*Culex* species and particularly mosquitoes of the *Culex pipiens* complex, are considered vectors of several diseases such as Lymphatic filariasis, West Nile Virus, Japanese Encephalitis, Saint Louis Encephalitis, Dengue and Rift Valley Fever with some being fatal in the absence of treatment and others causing lifelong disabilities and impairment^1^. In addition to their role as vectors, *Culex* species are also responsible for a high nuisance problem^2,3^. Indicatively *Culex* mosquitoes exhibit high biting rates exceeding 100 bites/person/night^2,4^. The *Cx. pipiens* complex member *Culex quinquefasciatus* is a prominent vector species which feeds on both humans and animals^5,6^, increasing its implication in pathogen transmission to both host groups. The vector displays a variety of breeding habitats including swamps, drains, pit latrin and permanent or semipermanent stagnant water bodies full of organic matters^7,8^, commonly found within and around African cities. Notably, the rapid unplanned urbanization of major cities in Africa, has favoured the installation of *Culex quinquefasciatus* within the urban environment^2,9^, including the cities of Yaoundé and Douala in Cameroon^2,8,10^.

Despite a growing interest in the promotion of integrated vector control strategies co-targeting different vector species, in Cameroon control efforts and relative entomological, epidemiological and insecticide resistance studies primarily focus on anophelines resulting in important knowledge gaps regarding *Culex* species and their control. In Cameroon, vector control mainly relies on the use of long-lasting insecticidal nets (LLINs) with over 35 million LLINs having been freely distributed to the population during mass distribution campaigns over the last decade^11^. This massive deployment of insecticide treated nets across the country and the increased use of pesticides in urban agriculture are considered to have driven insecticide resistance in *Anopheles* species which are now resistant to all insecticide compounds used in public health^12–15^. Indicatively, recent studies conducted in the city of Yaoundé reported a rapid expansion of insecticide resistance in *Anopheles gambiae* and *An. coluzzii* associated with different underlying mechanisms^12,13,16,17^. As *Culex* mosquitoes are usually found in sympatry with anophelines in sub-Saharan Africa^7,18^, it is possible that local *Culex* populations have also received high insecticide selection pressures^7,18^, driving the development of resistance in important vector species. A recent study on *Culex* mosquitoes from Cameroon points to this direction as phenotypic resistance to permethrin (mortality rate (mr) ranging from 14.25 to 66.05%) deltamethrin (mr: 2.91% to 20.78%), DDT (mr: 8.87% to 27.91%) and bendiocarb was recorded in the analysed populations^8^.

Insecticide resistance is primarily mediated by two molecular mechanisms: target site mutations—altering the insecticide’s target molecule and metabolic resistance—involving the increased activity of detoxification enzymes^19,20^. The voltage gated sodium channel (*VGSC*) gene point mutations L1014F, L1014S and L1014C (*kdr* mutations) associated with pyrethroid and secondarily organochlorine resistance in different insects have been recorded in a number of *Culex* populations across the world. Mutation L1014F, which upon homozygosity has been associated with operationally significant resistant phenotypes and striking ones, when in combination with P450 metabolic resistance^21^ has been recorded in *Cx. quinquefasciatus* populations from Benin, Cameroon and Zambia^8,22,23^ at allele frequencies ranging from 20 to 60%^24^.

The G119S mutation in the synaptic acetylcholinesterase gene, associated with organophosphate and carbamate resistance^25,26^ has also been recorded in African *Culex pipiens* complex populations, specifically from Morocco, Uganda and Benin albeit at low frequencies and mainly in heterozygosis^23,27–29^. G119S mutation has a high fitness cost and duplicated alleles have evolved on several occasions in *Culex* field populations creating a permanent state of ‘heterozygosity’, alleviating this cost^26,30,31^.

Detoxification enzymes belonging to the esterase and cytochrome P450 monooxygenases families have been associated with insecticide resistance in *Culex* mosquitoes. Overexpression of the P450 enzymes *CYP9M10*, *CYP6AA7*, *CYP6Z10*, *CYP4H34* and *Esterase A*, *Esterase B* have been associated with pyrethroid and organophosphate/carbamate resistance respectively in *Cx. quinquefasciatus* mosquitoes from Saudi Arabia, USA and the Western Indian Ocean Islands^20,30^. To date, there are very few studies investigating metabolic resistance in *Culex* mosquitoes from Africa, albeit not at the molecular level. For example, synergist bioassays on *Cx. pipiens* populations from Tanzania and Zanzibar^28,31^ implicate the involvement of monooxygenases, esterases, and glutathione S-transferase in pyrethroid and DDT resistance.

A third resistance mechanism, described in *Anopheles* species, involves cuticular alterations resulting in reduced insecticide uptake^32^. Cuticle thickening has also been reported in *Culex pipiens pallens* lab strains^33^ yet no field *Culex* populations have been analyzed for this resistance trait.

In light of the rapid expansion of insecticide resistance in mosquito vector populations in Cameroon and the limited knowledge on the local *Culex quinquefasciatus* resistance status the present study was undertaken (i) to assess the pattern of insecticide resistance in *Cx. quinquefasciatus* populations from Yaoundé against different insecticide classes and (ii) identify possible incipient resistance (i.e. resistant alleles at low frequencies) and investigate the underlying molecular mechanisms driving this resistance (i.e. target site, metabolic, cuticular).

## Results

### Species identification

To confirm morphological identification, a subsample of 40 mosquitoes were genotyped. All mosquitoes processed from the four study sites (Mendong, Nkolbisson, Tongolo and Etam-Bafia) were identified as *Cx. quinquefasciatus*.

### Susceptibility status of *Culex quinquefasciatus* populations

A total of 1,797 field-collected, 500 S-lab laboratory strain *Culex quinquefasciatus* specimens and 500 *An. gambiae* Kisumu strain were tested. WHO tube bioassays conducted with adult females, revealed high phenotypic insecticide resistance to pyrethroids, DDT and bendiocarb in *Cx. quinquefasciatus* from the four study sites (Table 1). No mortality was recorded when mosquitoes were exposed to 0.75% permethrin. Mortality rates ranging from 0 to 1.16% was recorded for 0.05% deltamethrin, 0–3% for 4% DDT and 0–14.63% for 0.1% bendiocarb. Resistance to malathion was observed in mosquitoes from Nkolbisson displaying a mortality rate of 57.5%. Mortality to malathion in the remaining populations ranged from 88 to 99%. The *An. gambiae* Kisumu strain was susceptible to all insecticides whereas the *Cx. quinquefasciatus* S-lab reference strains showed increase tolerance to permethrin and deltamethrin and high resistance to DDT.

**Table 1:** Susceptibility level of four *Culex quinquefasciatus* populations to 4% DDT, 0.75% permethrin, 0.05% deltamethrin, 0.1% bendiocarb and 5% malathion.

Mosquito population	Location	Insecticides	N tested	% mortality [95% CI]	Resistance status
Etam-Bafia	City centre	4% DDT	91	0	R
		0.75% permethrin	92	0	R
		0.05% deltamethrin	86	1.16 [− 1.10–3.42]	R
		0.1% bendiocarb	92	0	R
		5% malathion	100	88 [81.63–94.37]	R
Mendong	Periphery	4% DDT	100	0	R
		0.75% permethrin	78	0	R
		0.05% deltamethrin	80	0	R
		0.1% bendiocarb	82	14.63 [6.98–22.28]	R
		5% malathion	82	98.78 [96.41–101.15]	S
Nkolbisson	Periphery	4% DDT	88	0	R
		0.75% permethrin	80	0	R
		0.05% deltamethrin	82	0	R
		0.1% bendiocarb	83	1.2 [− 1.14–3.54]	R
		5% malathion	80	57.5 [− 46.67–68.33]	R
Tongolo	City centre	4% DDT	100	3 [− 0.34–6.34]	R
		0.75% permethrin	100	0	R
		0.05% deltamethrin	100	0	R
		0.1% bendiocarb	101	2.97 [− 0.37–6.31]	R
		5% malathion	100	89 [82.87–95.13]	R
Cx. Slab	Laboratory strain	4% DDT	300	13	/
		0.75% permethrin	300	82	/
		0.05% deltamethrin	300	90	/
		0.1% bendiocarb	100	99	/
		5% malathion	100	100	/
Kisumu strain (*An. gambiae*)	Laboratory strain	4% DDT	100	98	/
		0.75% permethrin	100	100	/
		0.05% deltamethrin	100	100	/
		0.1% bendiocarb	100	100	/
		5% malathion	100	100	/

R : resistant, S : susceptible.

### Intensity of pyrethroid resistance in *Culex quinquefasciatus* populations

**Figure 1:**
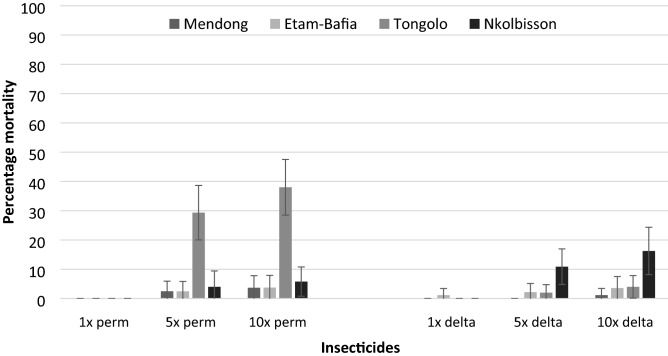
Resistance intensity of *Culex quinquefasciatus* populations from different districts of Yaoundé exposed to different concentrations (1 ×, 5 × and 10 ×) of permethrin and deltamethrin. Error bars represent 95% confidence intervals. Perm = permethrin, delta = deltamethrin.

High pyrethroid resistance intensity was recorded in *Culex quinquefasciatus* populations from all four study sites (Fig. 1). The mortality rate of *Cx. quinquefasciatus* was found to increase with the concentration of both permethrin and deltamethrin. Depending on the site, the mortality rate varied from 2.46 to 29.34% for permethrin 5 × and from 3.75 to 38% for permethrin 10 ×. For deltamethrin, the mortality rate varied from 0 to 2.17% for deltamethrin 5 × and from 1.17 to 16.25% for deltamethrin 10 ×.

### Effects of PBO synergist on the susceptibility of *Culex quinquefasciatus* to pyrethroids

**Figure 2:**
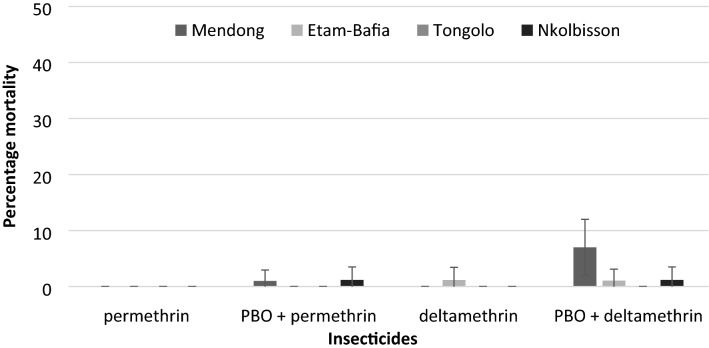
Effects of pre-exposure to 4% PBO on the susceptibility level of *Culex quinquefasciatus* to permethrin (1 ×) and deltamethrin (1 ×) in the city of Yaoundé. Error bars represent 95% confidence intervals.

Pre-exposure to PBO synergist significantly increased the susceptibility level of the Mendong *Cx. quinquefasciatus* population against deltamethrin, reporting a mortality rate of 7% (Fig. 2). Pre-exposure of the other populations to PBO did not alter the susceptibility levels against deltamethrin or permethrin.

### Screening of target site mutations (*kdr* L1014F, L1014S, L1014C, and *ace-1* G119S)

Mosquitoes resistant (bioassay survivors) to permethrin, deltamethrin or DDT were screened for *kdr* mutations at the 1014 *VGSC* locus. In Mendong, L1014F mutant allele frequency (MAF) reached 73.91%, in Nkolbisson 56.25%, in Tongolo 77.27% and in Etam-Bafia 55.00% (Table 2). The L1014S and L1014C *kdr* alleles were not detected in any of the samples.

**Table 2:** Distribution of the *kdr* L1014F and *ace-1* G119S alleles in different *Culex quinquefasciatus* populations from the city of Yaoundé.

Population	Resistant mutation allelic frequencies (hetero/homo)			
	Pyrethroids /DDT		Carbamates / Organophosphates	
	Sample size (alleles)	% *kdr* L1014F	Sample size (alleles)	% *ace-1* G119S
Mendong	46	73.91% (10/12)	48	0.00% (0/0)
Nkolbisson	48	56.25% (5/11)	60	33.33% (20/0)
Tongolo	22	77.27% (3/7)	92	19.56% (18/0)
Etam-Bafia	20	55.00 (3/4)	92	18.48% (17/0)

The *ace-1* G119S mutation was also detected in mosquito specimens resistant to malathion or bendiocarb. Apart from Mendong, where G119S mutation was not detected, subsamples of the three remaining locations display the mutation with a MAF varying from 18.48 to 33.33% (Table 2).

### Detoxification gene expression analysis

**Figure 3:**
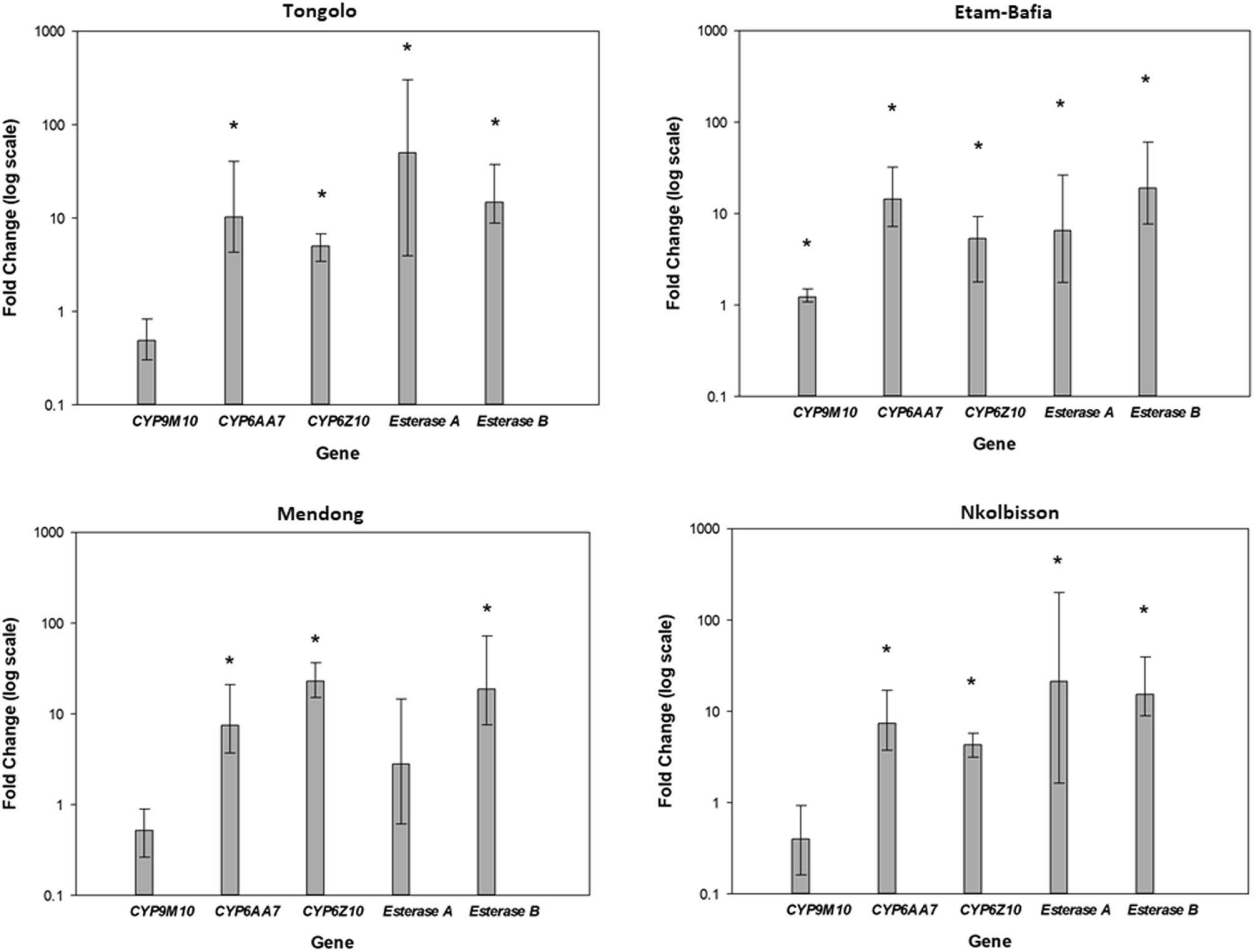
Expression analysis of detoxification genes in the four resistant *Culex quinquefasciatus* mosquito populations. Error bars indicate 95% CIs. Stars denote statistically significant upregulation.

The expression levels of six major detoxification genes associated with *Culex* metabolic resistance were compared between field *Cx. quinquefasciatus* populations and the S-lab susceptible laboratory strain. Detoxification genes analysed included *CYP9M10*, *CYP6AA7*, *CYP6Z10*, *CYP4H34*, *Esterase A* and *Esterase B*. The cytochrome P450 genes *CYP6AA7* and *CYP6Z10* were found to be upregulated (> 4.0 folds, *P* < 0.05) in all study sites. The *CYP9M10* was not strongly overexpressed in any of the study populations*.* The expression of *CYP4H34* gene was not detectable in any population, despite several attempts with alternative sets of primers. *Esterase B* was found to be overexpressed (> 10-folds) in all study sites and *Esterase A* in 3 of the 4 study sites (upregulation > 10-folds) (Fig. 3 and Suppl Table S2).

More precisely, the Mendong population showed a 7.54-fold and a 23.1-fold overexpression of *CYP6AA7* and *CYP6Z10*, respectively. The *Esterase B* gene was also found to be significantly overexpressed in Mendong (18.7-folds) (Fig. 3 and Suppl Table S2). In Nkolbisson, the *CYP6AA7* and *CYP6Z10* genes were overexpressed 7.46- and 4.32- times, respectively, along with both *Esterase* genes (21.3-folds for *Esterase A* and 15.4-folds for *Esterase B*) (Fig. 3 and Suppl Table S2). In Tongolo, *CYP6AA7* was overexpressed 10.24 times and *CYP6Z10* 5.01 times. *Esterase A* gene showed a 50.4-fold upregulation and *Esterase B* a 14.8-fold upregulation (Fig. 3 and Suppl Table S2). In the Etam-Bafia population a particularly robust overexpression of *CYP6AA7* was detected (14.5 folds), followed by *CYP6Z10* (5.36 folds). *Esterase A* and *Esterase B* genes were also overexpressed in Etam-Bafia (6.54- and 19.0- times, respectively) (Fig. 3 and Suppl Table S2).

### Analysis of cuticular hydrocarbon as a marker of reduced penetration based insecticide resistance

**Figure 4:**
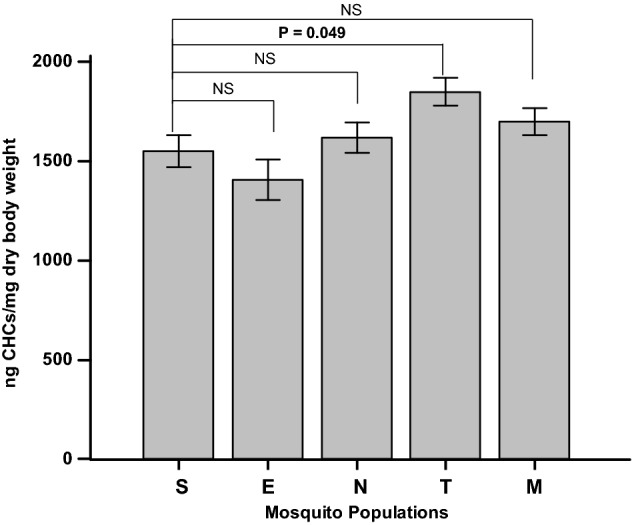
Mean CHC amounts from the five *Culex* mosquito populations. N and M mosquitoes have higher amounts of CHCs compared to S mosquitoes normalized for their size differences, but not E specimens. T mosquitoes have significantly higher CHCs compared to the S laboratory strain. Error bars: Standard Error of mean. S: Susceptible laboratory strain, E: Etam-Bafia, N: Nkolbisson, T: Tongolo, M: Mendong. NS: Not Statistically Significant.

Analysis of CHCs showed a statistically significant increase (*P* = 0.049) of normalised CHC content in the Tongolo population (1849 ± 70.0 ng CHCs/mg dry weight) compared to the susceptible laboratory strain (1552 ± 80.1 ng CHCs/mg dry weight). The remaining populations did not show a significant quantitative increase in their CHC profiles (Fig. 4).

## Discussion

Although *Culex quinquefasciatus* mosquitoes are predominant in most cities across Sub-Saharan Africa and they are of major epidemiological significance as vectors of important diseases like West Nile Virus and filariasis, little is known about their susceptibility to insecticides and particularly the presence and frequency of previously characterised resistance alleles in the local populations. The study objective was to assess the resistance status of *Cx. quinquefasciatus* populations from the city of Yaoundé against different insecticides.

High phenotypic resistance against DDT, deltamethrin, permethrin, bendiocarb, and malathion was detected in the analyzed *Cx. quinquefasciatus* populations.

The generated results are in line with previous bioassay records in *Culex* populations from the city of Yaoundé including *Culex quinquefasciatus, Cx. duttoni, Cx. antennatus, Cx. perfuscus* and *Cx. tigripes* specimens^8^. However here we report a significant decrease in the mortality rates of the tested Yaoundé populations, compared to the mortality rates recorded in previous studies^8^, indicating an ongoing process of insecticide resistance evolvement during the years 2017–2020.

Importantly, the tested populations from the four study sites of Yaoundé exhibited very high resistance intensity against permethrin and deltamethrin with mortality rates below 40% even when mosquitoes were exposed to the 10 × diagnostic concentration. This particularly low level of susceptibility to pyrethroids is of major concern with potentially operationally relevant implications in the control and elimination of diseases transmitted by these vectors.

The current expansion of pyrethroid and DDT resistance in *Cx. quinquefasciatus* may have derived from the increased selection pressure induced by the massive deployment of pyrethroid treated nets across the country in conjunction with the use of pyrethroid-based insecticides in agricultural pest control^11^.

Notably, the observed *Cx. quinquefasciatus* resistance profile was actually similar to that of *An. gambiae* populations from Yaoundé^12,13,16^ most likely attributed to common insecticidal pressures imposed on both vector species.

The *Culex quinquefasciatus* populations also displayed high resistance to both bendiocarb and malathion apart from the Mendong population which was susceptible to malathion. This is the first report of malathion resistance reported in *Culex* populations from Yaoundé.

Presence/frequency analyses of known target-site resistance mutations recorded the *kdr* allele L1014F at high frequencies in all populations. The recorded frequencies (ranging from 55–77%) were relatively increased to those reported in a previous study from Yaoundé city (frequency = 51%) conducted in 2017^8^ indicating an ongoing pyrethroid induced selection process.

The G119S *ace-1* mutation associated with both carbamate and organophosphate resistance in *Cx. quinquefasciatus* and *An. gambiae* sl.^26,42,43^ was also recorded in the analysed mosquitoes apart from the Mendong population. G119S was always recorded in heterozygosity, possibly due to the amplification events and fitness cost of this gene in the homozygous state^26,44,45^, while the non-detection of G119S in Mendong is in line with the respective population’s organophosphate susceptibility.

Use of the PBO synergist increased the deltamethrin susceptibility level of *Cx. quinquefasciatus* mosquitoes in some cases, indicating metabolic resistance and P450 monooxygenase enzyme might be involved in pyrethroid resistance.

In order to molecularly profile metabolic resistance, as a more specific and sensitive marker of metabolic resistance (including incipient resistance), the expression pattern of six detoxification genes (*CYP9M10*, *CYP6AA7*, *CYP6Z10*, *CYP4H34*, *Esterase A* and *Esterase B)* previously reported to be associated with insecticide resistance in *Cx. quinquefasciatus*^41,46–48^ were assessed in the four study populations.

The expression levels of two of the four cytochrome P450s analysed, *CYP6AA7* and *CYP6Z10,* were found to be upregulated (> 5.0 folds) in all study sites. The *CYP6AA7* gene has been shown to be overexpressed in pyrethroid resistant *Cx. quinquefasciatus*^41^ and is known to metabolize permethrin and its metabolites *in vitro*^49^ while *CYP6Z10* is also considered to be involved in pyrethroid resistance in *Cx. quinquefasciatus*^41^. Although *CYP4H34* and *CYP9M10* genes are known pyrethroid metabolisers^41^ their non-expression or non- biologically relevant overexpression in the examined populations could derive from the fact that they are predominantly expressed at the larval stage and show negligible expression in adults mosquitoes^41,50,51^. Under the prism of P450 target site synergism^21^ the co-presence of L1014F and *CYP6AA7, CYP6Z10* overexpression in the study populations may explain the strong resistance phenotypes against the pyrethroid insecticides.

*Esterases A and B* were found to be significantly overexpressed (> 10-folds, *P* < 0.05) in almost all populations. Esterases are considered to be implicated in organophosphate and carbamate resistance in *Culex* mosquitoes^52–54^ while they have also been reported to confer pyrethroid resistance in mosquitoes^31,52^ and in *Helicorverpa armigera*^55^.

Finally, a significant increase of CHC was detected in one study population possibly indicating that cuticle resistance may also contribute to the resistance phenotype of *Cx. quinquefasciatus* mosquitoes, in line with previous studies in *Anopheles species*^32,56^.

The multiple insecticide resistance mechanisms observed in *Cx. quinquefasciatus* populations from Yaoundé suggest high selective pressure taking place in this urban environment. In addition to insecticides, xenobiotics could also induce high resistance in this mosquito species due to its preference for organically polluted habitats at the larval stage^57^. The influence of organic pollution on *Cx. quinquefasciatus* susceptibility to insecticide has so far not been explored and requires further investigations.

## Conclusion

This study revealed the high resistance profile of *Cx. quinquefasciatus* populations from Yaoundé to DDT, permethrin, deltamethrin, bendiocarb and malathion. Several underlying mechanisms including the target site *kdr* mutations L1014F and *ace*-1 G119S, overexpression of cytochrome P450s and esterases A and B and possibly cuticle resistance were found to be associated to this resistance, in certain populations*.* The multi-resistance observed in the *Cx. quinquefasciatus* populations could affect the efficacy of insecticide interventions, thus the development of appropriate evidence-based insecticide resistance management programs under an integrated vector control approach is recommended.

## Methods

### Study sites

**Figure 5:**
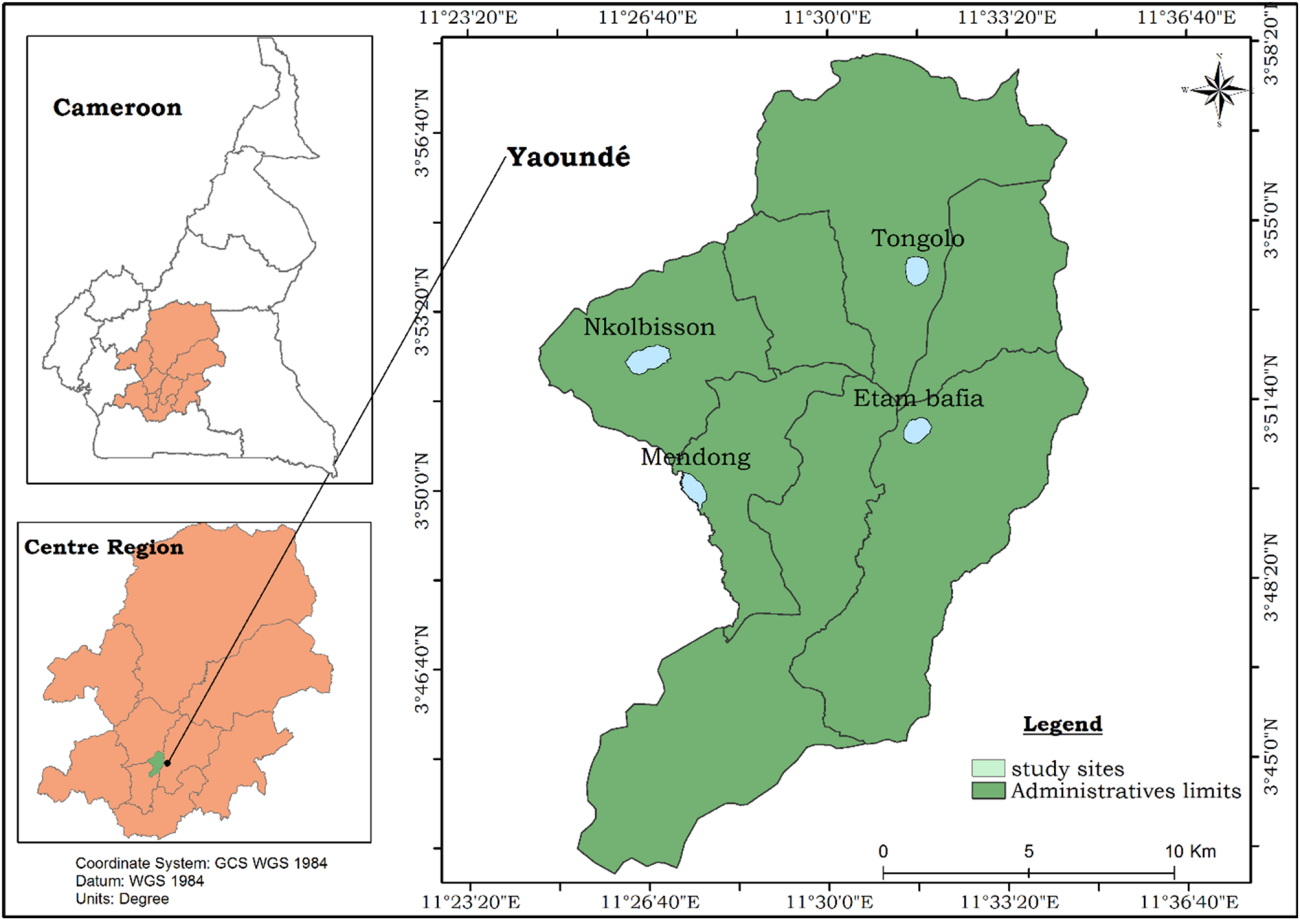
Study sites in the city of Yaoundé, Cameroon. The administrative division of Cameroon is available in open access on the OpenStreetMap platform (https://www.openstreetmap.org/search?query=cameroon#map=6/7.406/12.283). We used the ArcGIS version 10.2.2 software (ESRI, Redland, CA, USA; https://www.esri.com/en-us/arcgis/about-arcgis/overview) to generate the map showing study sites in Yaoundé.

The present study took place in Yaoundé (3° 51′ N; 11° 31′ E), the capital city of Cameroon. Yaoundé is located within the Congo-Guinean phytogeographic zone characterized by a typical equatorial climate with four seasons: two rainy seasons (March to June and September to November) and two dry seasons (December to February and July to August). The city has a population estimated at about 3 million inhabitants and is situated 800 m above sea level^34^. The landscape of Yaoundé is characterized by an alternation of high and lowland areas frequently used for agricultural practices. Mosquitoes were collected from September to November 2019 in four districts of Yaoundé, two situated in the periphery (Nkolbisson & Mendong) and two in the city centre (Tongolo & Etam-Bafia) (Fig. 5). Nkolbisson and Mendong are located along the Mefou river. They are densely populated areas with constructions extending to the swampy area and with the edges of the Mefou river exploited for the practice of agriculture during the dry season^35^. Tongolo and Etam-Bafia are two districts situated in the city centre. They are highly populated areas and characterized by poor drainage, high pollution and the presence of numerous standing water collections full of organic matter.

### Mosquito collection, rearing and processing

Blood fed *Cx. quinquefasciatus* females collected indoors with mouth aspirators were identified morphologically to species^36,37^ and maintained for rearing at the insectary. All blood fed females were given access to 10% sugar solution and were maintained at 25–30 °C and 70–80% relative humidity until they became fully gravid. The produced eggs were pooled and reared in trays containing dechlorinated water. After hatching larvae were fed using Tetramin baby fish food.

After emergence, unexposed, non-blood fed females aged 3–5 days from each site were divided into three batches. The first batch consisting of 50–60 specimens was preserved in RNA later for characterization of detoxification resistance mechanisms. The second batch comprising of 100–120 specimens was killed (after few minutes in the freezer) and air-dried at room temperature for 48 h for characterization of cuticular resistance. The remaining set of mosquitoes consisting of 500–700 specimens was used in bioassay experiments. Bioassay survivors against all insecticides were preserved in 70% ethanol and used for molecular species identification and detection of target-site mutations.

### Insecticide susceptibility tests

Bioassays were performed following the WHO guidelines^38^ with insecticides from four classes. For each mosquito population, four replicates of 20–25 F1 females each, were exposed to 0.75% permethrin, 0.05% deltamethrin, 4% DDT, 0.1% bendiocarb and 5% malathion. For each bioassay, two replicates of 20–25 female mosquitoes unexposed to any insecticide were used as an internal control. The reference *Culex* strain S-lab and *An. gambaie* Kisumu strain were tested alongside the field populations of *Cx. quinquefasciatus* to validate the efficacy of the impregnated papers. After 60 min exposure, mosquitoes were transferred to holding tubes and supplied with 10% glucose. The mortality rate was recorded 24 h post-exposure.

### Estimating insecticide resistance intensity

To estimate the intensity of pyrethroid resistance, mosquitoes were exposed to discriminating concentrations of permethrin and deltamethrin. For each test, 4 replicates of 20–25 females aged 3–5 days were exposed to the following concentrations: 0.05% (1 ×), 0.25% (5 ×) and 0.5% (10 ×) deltamethrin, and 0.75% (1 ×), 3.75% (5 ×) and 7.5% (10 ×) permethrin for 60 min. Mortality was recorded after 24 h. These bioassays were conducted following the WHO protocol described above^38^.

### Synergist bioassay

To assess the implication of P450 detoxification enzymes in pyrethroid resistance, bioassays with 4% PBO^39^, were conducted. For each mosquito population, four replicates of 20–25 F1 females mosquitoes were pre-exposed to PBO for 1 h and then transferred to tubes containing either 0.75% permethrin or 0.05% deltamethrin for 1 h. Bioassays were performed following the WHO protocol^38^. For each experiment, two replicates of 20–25 females mosquitoes exposed to PBO only were used as a control.

### Genomic DNA and total RNA extraction

Genomic DNA was extracted from individual mosquitoes using the DNazol protocol, according to the manufacturer’s instructions (Molecular Research Center Inc). Total RNA was extracted from pooled mosquito specimens (N = 10 per pool) using the TRI reagent (TR 118, Molecular Research Center Inc), following the manufacturer’s instructions. The quantity and purity of DNA and total RNA were assessed spectrophotometrically (Nanodrop). The quality of RNA was assessed by 1.0% w/v agarose gel electrophoresis.

### Identification of *Culex* species

Molecular identification of species was performed by applying the PCR-based assays for the identification of members of the *Cx. pipiens* complex described by Smith & Fonseca^40^. A total of 40 mosquitoes (10 from each collection site) was analysed.

### Detection of L1014 *kdr* mutations

The presence/frequency of L1014 *kdr* mutations was analyzed via Sanger Sequencing. The PCRs were carried out in 25 µl reactions containing 1 unit of Kapa Taq DNA polymerase (Kapa Biosystems), 0.4 mM dNTPs,1 × KapaTaq Buffer A (containing 1.5 mM MgCl_2_) and 0.3 µM each of the forward and reverse primers. The amplification consisted of an initial heat activation step at 95 °C for 5 min, followed by 40 cycles of 95 °C for 30 s, 54 °C for 30 s and 72 °C for 30 s with a final extension step at 72 °C for 5 min. The sequences of the primers used were F: 5′-TGATTGTGTTCCGGGTGCTG-3′ and R: 5′-GCAATTGCACCTTTAGGTGTGG-3′. The PCR fragments 350 bp were purified using NUCLEOSPIN Gel and PCR Clean-up (Macherey–Nagel). Nucleotide sequences were determined in purified PCR products at the GeneWiz sequencing facility (Leipzig, Germany).

### Detection of ACE-1 G119S mutation

Mosquitoes were tested for insensitive acetylcholinesterase (*ace*-1) mutations using the PCR–RFLP method as described in Weill et al.^26^. PCR products were digested with AluI (Minotech Biotechnology, Heraklion, Greece) restriction enzyme for 3 h and migrated on a 2.0% agarose gel.

### Gene expression analysis of major detoxification genes (CYP9M10, CYP4H34, CYP6AA7, CYP6Z10, Esterase A and Esterase B)

The expression analysis of major detoxification genes was done via reverse transcription and qPCR based on SYBR Green chemistry. cDNA was synthesized using 1 μg οf total RNA, previously treated with TURBO DNase (Invitrogen, Carlsbad, CA, USA), with oligo (dT)12–18 primers and the Minotech RT system kit (Minotech Biotechnology, Heraklion, Greece), following the manufacturer’s instructions. The SYBR Green-based qPCR assays were run in duplicates in 10 μl reactions, consisting of 2 × Kapa SYBR Fast Universal qPCR Master Mix (Kapa Biosystems, Wilmington, MA, USA), forward and reverse primers specific for each gene (Suppl Table S1) at a final concentration of 200 nM as well as 10 ng of cDNA template. Ribosomal Protein L8 (*RPL8*) and Ribosomal protein subunit 3 (*RPS3*) were used for normalization purposes. Bio-Rad CFX CONNECT Real-Time PCR detection was used with a thermal protocol consisting of a 3 min polymerase activation/initial denaturation step at 95 °C, 40 cycles of denaturation and annealing/extension steps at 95 °C for 3 s, 60 °C for 30 s, followed by a melting curve analysis step. A no-template control was included in each qPCR run. All assays were previously checked for specificity (melting curve and agarose gel electrophoresis of PCR products) and reaction efficiency, dynamic range and linearity (standard curve experiments) and reproducibility (estimation of the coefficient of variation-CV by analysing a series of samples in different runs). Quality control data are provided in Suppl Table S1.

### Extraction and Quantification of Cuticle Hydrocarbon (CHC) Lipids

Mosquitoes were air-dried at room temperature for 48 h and then pooled (20–25 female mosquitoes/replicate, 3 replicates for each population/strain), the dry weight of each replicate was measured, and CHC analysis was performed by GC–MS and GC-FID as previously described^13,32^.

### Ethical clearance

All experimental protocols were approved by the Cameroon National Ethics Committee on Human health (ethical clearance N° 2016/11/832/CE/CNERSH/SP). The committee reviews.

aspects related to both human and animal and all methods were carried out in accordance with relevant guidelines and regulations.

### Data analysis

The insecticide susceptibility data was interpreted as follows: 98%-100% mortality indicates susceptibility, 90%-97% mortality suggests possible resistance that needs to be confirmed, < 90% mortality suggests resistance^38^. Calculation of fold-changes, 95% CIs and statistical significance was performed according to the Pfaffl method^41^. Graphs of metabolic gene expression were constructed with the SigmaPlot software (v12.0). Differences were considered statistically significant at *P* < 0.05.

## Supplementary Information

Supplementary Information.
