# Supplementary material for: High insecticide resistance mediated by different mechanisms in Culex quinquefasciatus populations from the city of Yaoundé, Cameroon
Source: Sci Rep. 2021 Apr 1;11:7322. doi: 10.1038/s41598-021-86850-7 (PMC8017000; doi:10.1038/s41598-021-86850-7)
Supplement: Supplementary file 1 — Supplementary Information. [file 41598_2021_86850_MOESM1_ESM.pdf]

High insecticide resistance mediated by different mechanisms in *Culex quinquefasciatus* populations from the city of Yaoundé, Cameroon

Abdou Talipouo[1,2]*, Konstantinos Mavridis[3], Elysée Nchoutpouen[1], Borel Djiappi-Tchamen[1,4], Emmanouil A. Fotakis[3], Edmond Kopya[1,2], Roland Bamou[1,4], Sévilor Kekeunou[2], Parfait Awono-Ambene[1], Vasileia Balabanidou[3], Sofia Balaska[3], Charles S. Wondji[5,6], John Vontas[3,7] and Christophe Antonio-Nkondjio[1,5]*

[1] Laboratoire de Recherche sur le Paludisme, Organisation de Coordination pour la lutte contre les Endémies en Afrique Centrale (OCEAC), Yaoundé B. P.288, Cameroun;

[2] Department of Animal Biology and Physiology, Faculty of Sciences, University of Yaoundé 1, P.O. Box 337, Yaoundé-Cameroon

[3] Institute of Molecular Biology and Biotechnology, Foundation for Research and Technology-Hellas, 70013 Heraklion, Greece

[4] Vector Borne Diseases Laboratory of the Research Unit Biology and Applied Ecology (VBID-RUBAE), Department of Animal Biology, Faculty of Science of the University of Dschang

[5] Vector Biology Liverpool School of Tropical medicine Pembroke Place, Liverpool L3 5QA, UK;

[6] Centre for Research in Infectious Disease (CRID), Yaoundé P.O. Box 13591, Cameroun

[7] Pesticide Science Laboratory, Department of Crop Science, Agricultural University of Athens, 11855 Athens, Greece

Email addresses

Talipouo A (* Corresponding author): atalipouo@gmail.com

Mavridis K: mavridiskos@gmail.com

Nchoutpouen E: enchoutpouen2002@yahoo.fr

Djiappi-Tchamen B: borel_tchamen@yahoo.com

Fotakis E: bio1763@gmail.com

Kopya E : edmondoev@yahoo.fr

Bamou R : bamou2011@gmail.com

Kekeunou S: skekeunou@gmail.com

Awono-Ambene P: hpaawono@yahoo.fr

Vasileia Balabanidou: balaban@imbb.forth.gr

Sofia Balaska: sofia_balaska@imbb.forth.gr

Charles S. Wondji: charles.wondji@lstmed.ac.uk

Vontas John: vontas@imbb.forth.gr

Christophe Antonio-Nkondjio (* Corresponding author): antonio_nk@yahoo.fr

Supplementary Table S1 Details of the qPCR assays that were applied for the gene expression analysis of major detoxification genes

Gene	Primers	Sequence	Reaction efficiency	R^2	%CV	Dynamic Range (Ct values)	Melting curve	Agarose gel electrophoresis
RPS3	F	AGCGTGCCAAGTCGATGAAG	97.32 %	0.9918	<10%	17.5-31.5	Single peak	Single band in the expected size
	R	ACGTACTCGTTGCACGGATCTC						
	R	GCATCAAGCGCCACCATATAGG						
RPL8	F	GCTGGCCGAAGGTGCGTGGT	91.3%	0.9985	<10%	14.8-30.0	Single peak	Single band in the expected size
	R	TTGCGACCTGGCGGCGTTCC						
CYP9M10	F	GCAAAGAGAAGAGCACAACATTG	105%	0.9987	<10%	23.0-32.3	Single peak	Single band in the expected size
	R	CTCCGTTGAGGACTGAAGATG						
CYP6Z10	F	AGCGGTTCTTCGAGGAAAGC	97.2%	0.9997	<10%	22.18-30.0	Single peak	Single band in the expected size
	R	GTTGGACAGCAGCATTATGAGG						
CYP6AA7	F	ATGACGCTGATTCCCGAGACTGTT	97.2%	0.9999	<10%	21.9-31.1	Single peak	Single band in the expected size
	R	TTCATGGTCAAGGTCTCACCCGAA						
Esterase A	F	GGCGCACTTGGTATGATATGTG	99.5%	0.988	<10%	27.3-32.7	Single peak	Single band in the expected size
	R	TCTGGTCCTTTAGTCCGGCA						
Esterase B	F	ACGGTCCGGATTTCTTGGTT	105%	0.9996	<10%	23.2-31.3	Single peak	Single band in the expected size
	R	TCCTGCACCGATTGACAACA						
CYP4H34	F1	CCGTTCAGCGTGGGATCG		N/A (Gene expression was not detectable)				
	R1	GCAGCACCAGGTCCATCTTG						

	F2	CATCCAGCTGGCAA AGCACC		
	R2	GACTTCTGCGCCGA GTACG		
	F3	TTTACGATGCTGTT GTTTGCGATTC		
	R3	CTGCACCCTAATCG GAATCC		
	F4	GCCGAACCCACCAA TCAGTA		
	R4	GGGTGGAAAATGC GCTTCAG		
	F5	CTCGGGCATTTCCTT CACCA		
	R5	TTGGTTAAGGGGTG CGGTTT		

Suppl Table S2 Overexpression fold changes of detoxification genes in four *Culex quinquefasciatus* mosquito populations compared to the S-lab susceptible mosquito strain. Bold letters indicate statistically significant upregulation.

Populations	Detoxification genes fold changes (95% CI), P value				
	CYP9M10	***CYP6AA7***	***CYP6Z10***	***Esterase A***	***Esterase B***
Mendong	0.519<br>(0.265 - 0.889)<br>P = 0.009	**7.54**<br>(3.72 - 21.1)<br>P = 0.002	**23.1**<br>(15.1 - 36.6)<br>P = 0.017	2.83<br>(0.611 - 14.6)<br>P = 0.144	**18.7**<br>(7.61 - 71.9)<br>P= 0.011
Nkolbisson	0.400<br>(0.162 - 0.926)<br>P = 0.004	**7.46**<br>(3.77 - 17.0)<br>P = 0.003	**4.32**<br>(3.16 - 5.79)<br>P = 0.005	**21.3**<br>(1.63 – 199)<br>P = 0.009	**15.4**<br>(8.91 - 39.5)<br>P = 0.012
Tongolo	0.486<br>(0.304 - 0.824)<br>P = 0.015	**10.24**<br>(4.34 - 40.5)<br>P = 0.014	**5.01**<br>(3.46 - 6.79)<br>P = 0.029	**50.4**<br>(3.94 – 304)<br>P = 0.018	**14.8**<br>(8.82 - 37.7)<br>P = 0.011
Etam-Bafia	**1.23**<br>(1.075 - 1.492)<br>P = 0.002	**14.5**<br>(7.20 - 32.1)<br>P = 0.002	**5.36**<br>(1.79 - 9.28)<br>P = 0.002	**6.54**<br>(1.77 - 26.4)<br>P < 0.001	**19.0**<br>(7.7 - 60.9)<br>P < 0.001

Note: CYP4H34 gene was also tested but no expression profile was detected.
